# Effectiveness of a Mobile eHealth App in Guiding Patients in Pain Control and Opiate Use After Total Knee Replacement: Randomized Controlled Trial

**DOI:** 10.2196/16415

**Published:** 2020-03-13

**Authors:** Yvette Pronk, Maud Cornelia Wilhelmina Maria Peters, Amarsing Sheombar, Justus-Martijn Brinkman

**Affiliations:** 1 Research Department Kliniek ViaSana Mill Netherlands; 2 Department of Anaesthesiology Kliniek ViaSana Mill Netherlands; 3 Department of Orthopaedic Surgery Kliniek ViaSana Mill Netherlands

**Keywords:** opiate use, pain, total knee replacement, app, eHealth

## Abstract

**Background:**

Little is known about pain and opiate use at home directly after total knee replacement (TKR). Due to adverse effects, low opiate use is desired. An electronic health app (PainCoach) was developed to guide patients in pain control and opiate use.

**Objective:**

The aim of this paper was to investigate the effects of the PainCoach app on pain control and opiate use in patients who underwent TKR during the first 2 weeks at home after surgery.

**Methods:**

In an unblinded randomized controlled trial, patients scheduled for TKR were offline recruited and randomized to a PainCoach group or control group. In the PainCoach group, the PainCoach app was downloaded on each patient’s smartphone or tablet. In response to the patient’s input of the pain experienced, the PainCoach app gave advice on pain medication use, exercises/rest, and when to call the clinic. This advice was the same as that received during usual care. The control group received usual care. The primary outcomes were opiate use and visual analog scale (VAS) pain scores at rest, during activity, and at night during the first 2 weeks at home after surgery, which were collected daily from day 1 until 14 postoperatively by online questionnaires. The actual amount of app use was recorded, and active use was defined as ≥12 total app uses.

**Results:**

The pain scores did not differ between the groups. The PainCoach group (n=38) used 23.2% less opiates (95% CI −38.3 to −4.4; *P*=.02) and 14.6% more acetaminophen (95% CI 8.2-21.3; *P*<.001) when compared with the findings in the control group (n=33). The PainCoach app was used 12 (IQR 4.5-22.0) times per patient. In the active PainCoach subgroup (n=19), the following were noted when compared with the findings in the control group: 4.1 times faster reduction of the VAS pain score during activity (95% CI −7.5 to −0.8; *P*=.02), 6.3 times faster reduction of the VAS pain score at night (95% CI −10.1 to −2.6; *P*=.001), 44.3% less opiate use (95% CI −59.4 to −23.5; *P*<.001), 76.3% less gabapentin use (95% CI −86.0 to −59.8; *P*<.001), and 21.0% more acetaminophen use (95% CI 12.6-30.0; *P*<.001).

**Conclusions:**

The use of the PainCoach app contributes to reduced opiate use in the initial period at home after TKR. Active use of this app leads to a further reduction in opiate use and improved pain control.

**Trial Registration:**

ClinicalTrials.gov NCT03961152; https://clinicaltrials.gov/ct2/show/NCT03961152

## Introduction

Total knee replacement (TKR) is a successful treatment option for patients with end-stage knee osteoarthritis (OA) [1]. Moderate-to-severe pain after TKR can be expected [2,3]. Local infiltration anesthesia (LIA) techniques and so-called fast-track recovery programs have resulted in reduced pain and early mobilization, subsequently reducing the length of stay in hospital and increasing patient satisfaction [4-7]. Previous research established several factors associated with increased pain after TKR [8-19] (Table 1). Postoperative pain inhibits recovery, increases morbidity, and may result in chronic pain, ultimately limiting the effectiveness of TKR [6,20]. Therefore, pain should be controlled optimally both in the hospital and at home.

**Table 1:** Factors associated with increased pain after total knee replacement.

Factor	Association with increased pain after TKR^a^
Gender	Being female [8-12]
Age	Older age [8,10,13]
BMI^b^	Higher BMI^b^ [8,10]
ASA^c^ score	Higher ASA^c^ score [10]
Pain catastrophization	Higher pain catastrophization score [12,14-17]
Comorbidity	Presence of comorbidities [8,10,13,18]
Previous knee surgery	Having a history of knee surgery [10]
Preoperative pain	Higher preoperative pain severity [8,12,18,19]
Social support	Poor social support [13]
Preoperative mental health	Poor preoperative mental health [8,10,13,18]

^a^TKR: total knee replacement.

^b^BMI: body mass index.

^c^ASA: American Society of Anesthesiologists.

Although pain is usually under control during hospital stay, less is known about pain control in the initial period at home after TKR. Current pain management strategies include a combination of nonsteroidal anti-inflammatory drugs (NSAIDs), nonnarcotic medication, opiates, and exercise [4]. Although opiates are very effective for reducing pain, serious adverse effects, such as nausea, itching, reduced gut mobility, and urinary retention, often occur [21]. Addiction to opiates is an ever increasing problem and may ultimately lead to an increased risk of death [22]. The amount of opiate use should therefore be kept to a minimum. Orthopedic surgery, however, accounts for an estimated 8.8% of prolonged prescription opiate use [23]. Therefore, alternative pain management strategies are needed. Electronic health (eHealth) apps can be used to guide patients in improving their pain management strategies at home. An important benefit of these apps is that patients can access the information provided directly and anywhere whenever necessary [24-29]. The number of older adults with internet access and acceptance of internet-based interventions is increasing, and patients tend to remember up to 80% of the information acquired from interactive education [30,31].

With this in mind, to manage pain better and potentially decrease opiate use, an eHealth app named PainCoach was developed. This app aims to help patients control their pain better in the initial period at home after TKR, including optimal use of the available pain medication. This study aimed to determine the effects of PainCoach on pain control and opiate use in TKR patients in the first 2 weeks at home after surgery. The hypothesis was that the use of this app would decrease pain and opiate use.

## Methods

### Study Design

An unblinded, randomized, controlled, single-center trial was performed at Kliniek ViaSana (Mill, The Netherlands). Patients with an American Society of Anesthesiologists (ASA) score of I-II, a body mass index (BMI) of ≤35, and a plan to undergo primary TKR between February and June 2016 were enrolled. Four experienced high-volume knee surgeons performed all surgeries, and three experienced anesthesiologists administered spinal anesthesia. The same type of TKR implant was used in all patients (NexGen LPS, ZimmerBiomet, Warsaw, Indiana). All surgeries were performed using a tourniquet. The pain management protocol consisted of preoperatively administered medication, LIA injections during surgery directly before cementing the implant, and a step-wise postoperative pain management protocol (Multimedia Appendix 1). Patients were excluded if they did not possess a smartphone or tablet, had a contraindication to any of the medications used in the study, did not have an email address, did not have internet at home, did not have a thorough command of the Dutch language, had memory disorders, or had surgery under general anesthesia. Patients were recruited over the phone by the research staff after being scheduled for primary TKR under spinal anesthesia, and contraindication to any of the medications used in the study and presence of memory disorders were checked by the anesthesiologists. Patients were asked over the phone if they possessed a smartphone or tablet, had an email address, had internet at home, and had a thorough command of the Dutch language. Patient information and informed consent were sent by postal service if a patient met the criteria and was interested to participate. Patients were considered lost to follow-up if they completed less than two postoperative questionnaires during the first 2 weeks at home. Power analysis (significance level: .05, power: 90%) showed that 35 patients would be needed in each group to detect a difference of 10 points on a visual analog scale (VAS) for pain (VAS pain, 0-100). Written informed consent was obtained from all participants. The study was approved by the medical ethics committee of St. Anna Hospital (Geldrop, The Netherlands, Study ID: 5.12) and was registered at Clinicaltrials.gov retrospectively (ID: NCT03961152).

### Randomization

Included unblinded patients were randomly assigned to the PainCoach or control group using lots presented in sealed opaque envelopes during admission. All lots were created and sealed by a researcher in the ratio of 1:1. A blinded nurse presented the envelops to a patient, and the patient selected one to complete randomization. All patients received the usual pain management care including pre-, peri-, and postoperative pain medication (Multimedia Appendix 1), participated in group information meetings, received an information booklet, and could contact the clinic at any time (24 hours a day/7 days a week) in case of any remaining questions. In the PainCoach group, in addition to receiving the aforementioned usual care, the PainCoach app (Interactive Studios, Rosmalen, The Netherlands) was downloaded on each patient’s smartphone or tablet, using a unique download code. In this way, the PainCoach app was not available to the control group. An unblinded nurse provided the code and assisted the patient by completing the download process of the app during admission. The app gave the same advice as that during usual care. After only entering the date of surgery as patient data, the app allowed patients to input their pain level (no pain, bearable pain, unbearable pain, or untenable pain) whenever they wanted until day 14 after surgery. Based on the patient’s input and taking into account the number of days after surgery, the app provided advice on pain medication use, physiotherapy exercises including videos, use of ice or heat packs, rest, immobilization of the operated leg, and when to call the clinic (Multimedia Appendix 2). Patients in the PainCoach group were not subjected to any treatment that was different from that in the control group (ie, advice on pain management was delivered in an extra and different way, but the pain medication itself was exactly the same for both groups). During the study, no major changes or revisions were made to the PainCoach app.

### Outcomes and Measurements

Beside the actual amount of app use, all the outcome measurements were assessed using a digital, online, automated collection system (OnlinePROMs, Interactive Studios, Rosmalen, The Netherlands), which automatically sent an invitation by email to complete an online questionnaire preoperatively, daily from day 1 to 14, and at 1 month postoperatively. In case of nonresponse to the preoperative or 1-month questionnaire, an automatic reminder was sent after 3 days. The invitation to complete the daily questionnaire was sent at 5 pm, and patients had access to the questionnaire until midnight.

The primary outcomes were opiate use and pain score of the operated knee at rest, during activity, and at night in the first 2 weeks at home after TKR. The pain score was measured on a VAS for pain, which ranged from 0 (no pain) to 100 (worst imaginable pain), preoperatively, daily from day 1 to 14, and at 1 month postoperatively [32-35]. Severe pain was defined as a VAS pain score from 70 to 100. Opiate (oxycodon; 5 mg per tablet; different manufacturers) use was recorded in quantities per 24 hours from day 1 to 14.

The secondary outcomes in the first 2 weeks at home and 1 month after TKR included other pain medication use (ie, NSAIDs [diclofenac], acetaminophen, or gabapentin; different manufacturers), which was also recorded in quantities per 24 hours from day 1 to 14. Additionally, pain acceptance at rest, during activity, and at night was assessed with a happy smiley (acceptable pain) and a sad smiley (unacceptable pain) preoperatively, daily from day 1 to 14, and at 1 month postoperatively. Experiences with the executed recommended physiotherapy exercises were recorded daily from day 1 to 14 on a 3-item scale (did too much, exactly enough, or could have done more exercises). Moreover, function and quality of life were measured preoperatively and 1 month postoperatively. Knee function was assessed using the Knee Injury and Osteoarthritis Outcome Score–Physical Function Short-form (KOOS-PS) on a scale from 0 (no difficulty) to 100 (extreme difficulty) [36]. The Oxford Knee Score was used to measure combined function and pain on a scale from 0 (most severe symptoms) to 48 (least severe symptoms) [37]. Quality of life was measured using the EuroQol-5 Dimensions (EQ-5D) 3-level version (EQ-5D-3L) questionnaire consisting of the following two scores: EQ VAS score, which is assessed on a scale from 0 (worst imaginable health state) to 100 (best imaginable health state), and EQ-5D descriptive system [38]. The PainCoach app’s perceived effectiveness (usability, added value, and likelihood of being recommended to others) was recorded on a 5-item scale ranging from totally agree to totally disagree at day 14 after surgery. Each downloaded app had its own app code that was used to record the actual amount of app use. As the admission period was generally 1 or 2 days, outcomes were measured until day 14 after surgery, and outcomes at home were investigated, the outcome active PainCoach app use was defined as using the app at least 12 times in total.

Preoperative opiate and other pain medication use, age, gender, ASA score, BMI, preoperative comorbidities, history of knee surgery on the same side, Charnley score, date of surgery, date of discharge, and complication data were collected from the electronic patient records. Pain coping, anxiety, education level, and marital status were determined preoperatively using an online questionnaire. Pain coping was measured using the pain coping and cognition list scored from 1 (totally disagree) to 6 (totally agree), and it had the following four categories: catastrophizing, pain coping, internal pain management, and external pain management [39].

### Statistical Analysis

Analysis was performed using SPSS version 25.0 (IBM Corp, Armonk, New York). All measured outcomes from day 1 until day 14 after surgery were recoded into measured outcomes for days at home by subtraction of the admission period. Patient characteristics were analyzed using descriptive statistics, and data were checked for normal distribution. Differences in mean, median, or percentage were tested using the independent two-sample *t*-test, Mann-Whitney *U* test, likelihood analysis, Fisher’s test, or Pearson’s chi-squared test, depending on the type of data. Mixed linear models were used to analyze the overall rate of decrease or increase for continuous data, and generalized linear models were used to analyze the percentage decrease or increase for count and nominal data. Additional analysis was performed to compare the active PainCoach subgroup with the control group, with correction for differences in preoperative data. Statistical significance was set at *P*<.05, and trends were defined as .05<*P*<.10.

## Results

### Patient Characteristics

**Figure 1:**
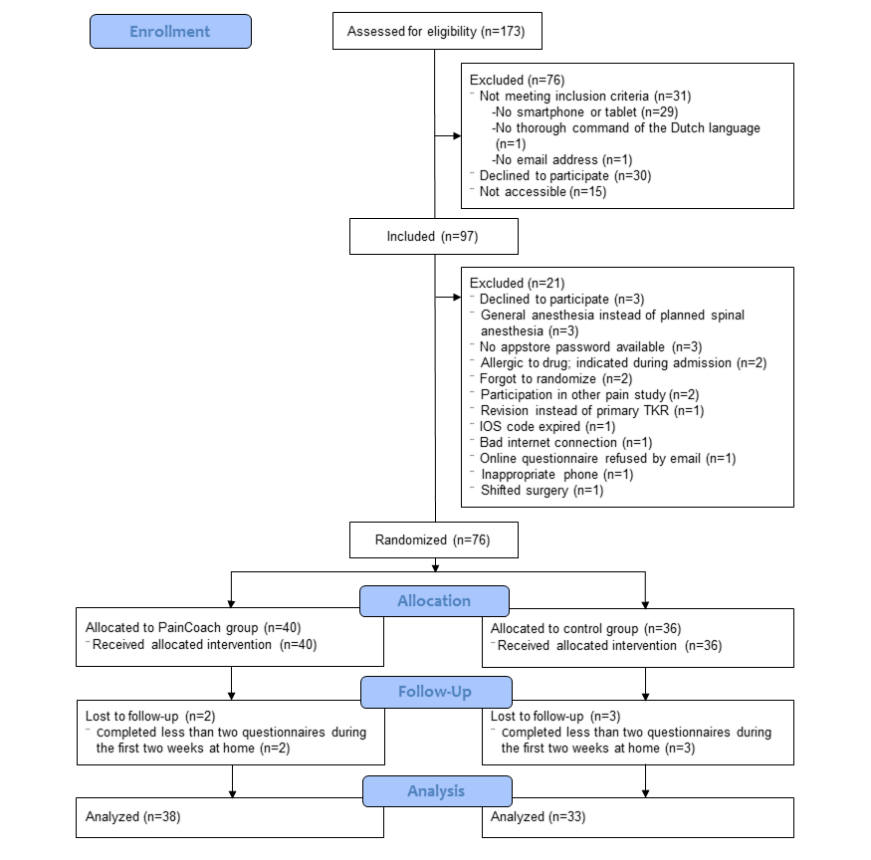
Study flowchart. IOS: iPhone operating system; TKR: total knee replacement.

A total of 97 patients were included, and of these, 76 patients were randomized. Because of loss to follow-up, the final analysis was performed with 71 patients (PainCoach group, n=38; control group, n=33) (Figure 1). The response rates for the daily questionnaires at home were 91% in the PainCoach group and 89% in the control group.

No statistically significant differences in patient characteristics were found between the PainCoach group and control group. The preoperative VAS pain score at night was significantly lower in the active PainCoach subgroup (n=19) than in the control group (*P*=.02) (Table 2).

**Table 2:** Characteristics of patients in the PainCoach group, active PainCoach subgroup, and control group.

Characteristic			1. PainCoach (n=38)	2. Active PainCoach (n=19)	3. Control (n=33)	*P* value (1 vs 3)	*P* value (2 vs 3)
Gender (male), n (%)			23 (61)	13 (68)	19 (58)	.80	.44
Age (years), mean (SD)			62.6 (7.0)	62.8 (6.1)	64.6 (7.5)	.24	.38
BMI^a^, mean (SD)			27.6 (3.5)	26.7 (3.4)	27.8 (3.0)	.83	.24
ASA^b^ (I), n (%)			18 (47)	11 (58)	12 (36)	.35	.13
Preoperative comorbidities, n (%)			14 (37)	8 (42)	17 (52)	.21	.51
**Preoperative prescription, n (%)**							
		NSAIDs^c^	5 (13)	3 (16)	6 (18)	.20	.48
		Acetaminophen	1 (3)	0 (0)	1 (3)	.92	>.99
		Opiate	3 (8)	0 (0)	0 (0)	.24	>.99
		Gabapentin	0 (0)	0 (0)	0 (0)	>.99	>.99
**Preoperative anxiety, n (%)**						.59	>.99
		No anxiety	33 (87)	18 (95)	30 (91)		
		Some anxiety	5 (13)	1 (5)	3 (9)		
		Much anxiety	0 (0)	0 (0)	0 (0)		
History of knee surgery on the same side, n (%)			27 (71)	15 (79)	21 (64)	.51	.25
**Charnley score, n (%)**						.64	.98
		One knee affected with OA^d^	22 (58)	12 (63)	19 (58)		
		Both knees affected with OA	7 (18)	3 (16)	6 (18)		
		Contralateral TKR^e^	5 (13)	1 (5)	2 (6)		
		Multiple joints affected with OA	4 (11)	3 (16)	6 (18)		
**Education level, n (%)**						.33	.30
		Primary school	3 (8)	1 (5)	1 (3)		
		Secondary school	14 (37)	7 (37)	10 (31)		
		Tertiary school	21 (55)	11 (58)	21 (66)		
**Marital status, n (%)**						.19	.09
		Married	29 (76)	18 (95)	22 (67)		
		Other^f^	9 (24)	1 (5)	11 (33)		
**Pain coping, mean (SD)**							
		Catastrophization	2.5 (0.7)	2.5 (0.8)	2.3 (0.6)	.15	.32
	Pain coping		3.6 (1.0)	3.8 (1.0)	3.7 (0.8)	.68	.66
	Internal pain management		4.1 (0.8)	4.2 (0.9)	3.9 (0.8)	.24	.29
	External pain management		2.7 (0.8)	2.7 (0.7)	2.5 (0.8)	.31	.36
**Preoperative VAS^g^ pain, median (IQR^h^)**							
		Knee at rest	33.0 (20.8-52.8)	33.0 (13.0-43.0)	32.0 (17.8-49.0)	.65	.82
		Knee during activity	60.5 (36.5-77.3)	57.0 (30.0-75.0)	60.0 (43.3-73.8)	.82	.69
		Knee at night	20.5 (4.8-42.5)	15.0 (1.0-30.0)	35.5 (15.0-58.5)	.11	.02^i^
**Preoperative acceptable pain, n (%)**							
		Knee at rest	29 (76)	16 (84)	27 (82)	.40	>.99
		Knee during activity	16 (42)	10 (53)	13 (39)	.90	.41
		Knee at night	28 (74)	16 (84)	27 (82)	.28	>.99
Preoperative KOOS-PS^j^, median (IQR)			47.3 (41.6-55.3)	46.1 (40.3-54.4)	48.5 (40.3-57.9)	.76	.96
Preoperative OKS^k^, mean (SD)			25.3 (7.2)	27.0 (7.2)	24.8 (5.6)	.75	.23
Preoperative EQ-5D^l^ descriptive system, median (IQR)			0.775 (0.471-0.783)	0.775 (0.516-0.807)	0.775 (0.651-0.807)	.27	.81
Preoperative EQ VAS^m^, median (IQR)			86.0 (73.6-94.3)	87.0 (79.0-93.0)	86.0 (74.0-95.5)	.89	.72
Complications, n (%)			3 (8)	2 (11)	1 (3)	.62	.55

^a^BMI: body mass index.

^b^ASA: American Society of Anesthesiologists.

^c^NSAIDs: nonsteroidal anti-inflammatory drugs.

^d^OA: osteoarthritis.

^e^TKR: total knee replacement.

^f^Other marital status: single, living together, divorced, widow(er), living apart together relationship, different.

^g^VAS: visual analog scale.

^h^IQR: interquartile range.

^i^Significant difference (*P*<.05).

^j^KOOS-PS: Knee Injury and Osteoarthritis Outcome Score–Physical Function Short-form.

^k^OKS: Oxford Knee Score.

^l^EQ-5D: EuroQol-5 Dimensions.

^m^EQ VAS: EuroQol visual analog scale.

### Visual Analog Scale Pain Scores and Opiate Use

**Figure 2:**
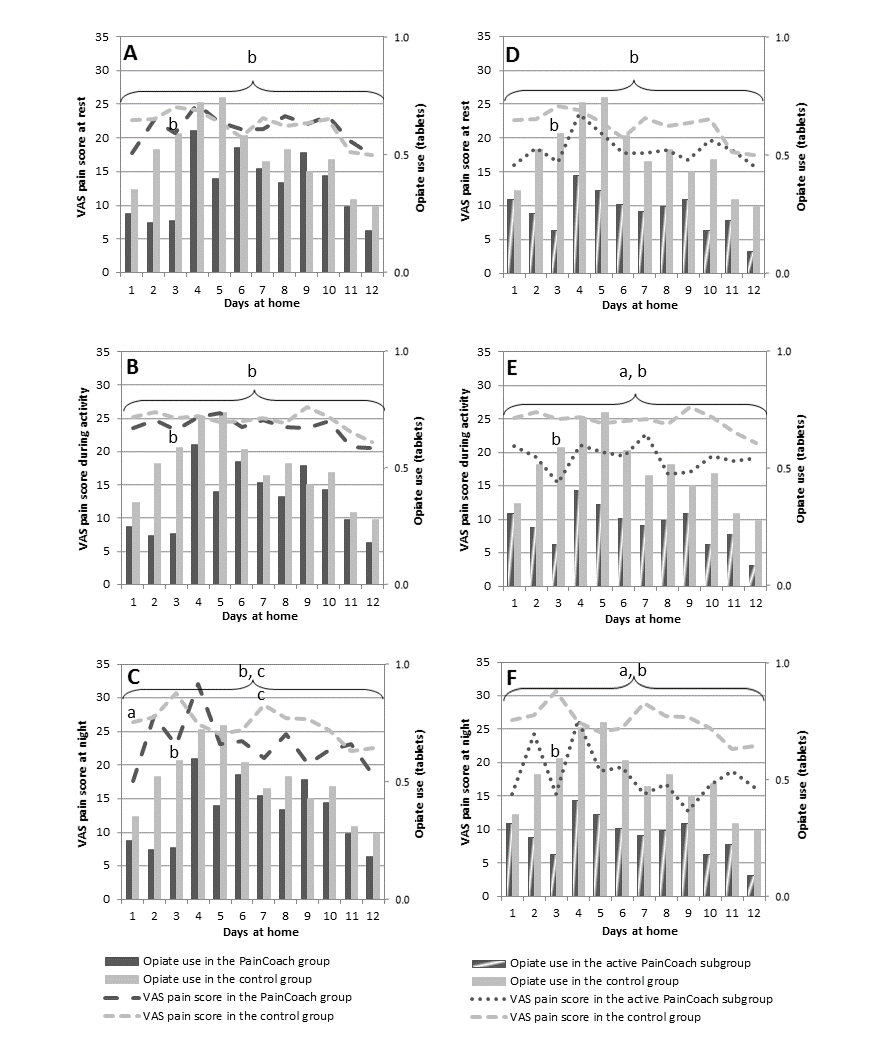
VAS pain scores and opiate use in the PainCoach group and control group at rest (A), during activity (B), and at night (C) and in the active PainCoach subgroup and control group at rest (D), during activity (E), and at night (F) on separate days and in the overall first period at home. a: significant difference in VAS pain (*P*<.05); b: significant difference in opiate use (*P*<.05); c: trend in VAS pain (.05<*P*<.10); VAS: visual analog scale.

During the first 2 weeks at home, the PainCoach group had VAS pain scores of 17.0 (IQR 5.0-30.0) at rest, 20.0 (IQR 7.0-35.0) during activity, and 17.0 (IQR 4.0-37.0) at night. The control group had VAS pain scores of 20.0 (IQR 7.0-33.0) at rest, 21.0 (IQR 10.0-38.0) during activity, and 20.5 (IQR 8.0-40.0) at night. Pain was classified as severe on one or more days in 21% (8/38) of patients from the PainCoach group and 30% (10/33) of patients from the control group. No statistically significant differences were found between the two groups in terms of the VAS pain scores at rest, during activity, and at night (Figure 2A-C, Table 3). Regarding opiate use, the PainCoach group used a mean of 0.4 (SD 0.7) tablets a day and the control group used a mean of 0.5 (SD 0.8) tablets a day. Opiate use was significantly reduced by 23.2% in the PainCoach group when compared with the finding in the control group (95% CI −38.3 to −4.4; *P*=.02) (Figure 2A-C, Table 3). One month after surgery, no statistically significant differences in the VAS pain scores were found between the PainCoach group and control group (Table 4).

Adjusted analyses showed that the active PainCoach subgroup had VAS pain scores of 10.0 (IQR 4.0-26.3) at rest, 12.0 (IQR 5.0-25.0) during activity, and 10.0 (IQR 2.8-28.0) at night during the first 2 weeks at home. Pain was reported as severe on one or more days in 16% (3/19) of patients from the active PainCoach subgroup. The VAS pain score during activity significantly decreased 4.1 times faster in the active PainCoach subgroup when compared with the finding in the control group (95% CI −7.5 to −0.8; *P*=.02) (Figure 2E, Table 3). The VAS pain score at night significantly decreased 6.3 times faster in the active PainCoach subgroup when compared with the finding in the control group (95% CI −10.1 to −2.6; *P*=.001) (Figure 2F, Table 3). The mean opiate use was 0.3 (SD 0.5) tablets a day in the active PainCoach subgroup. Opiate use was significantly reduced by 44.3% in the active PainCoach subgroup when compared with the finding in the control group (95% CI −59.4 to −23.5; *P*<.001) (Figure 2D-F, Table 3). One month after surgery, no statistically significant differences in VAS pain scores were found between the active PainCoach subgroup and control group (Table 4).

**Table 3:** Findings in the PainCoach group, active PainCoach subgroup, and control group during the first 2 weeks at home.

Variable		1. PainCoach versus control	2. Active PainCoach versus control	*P* value (1)	*P* value (2)
**VAS^a^ pain,** **decrease or increase (rate)**					
	Knee at rest	↓0.3	↓1.9	.86	.27
	Knee during activity	↓1.0	↓4.1	.48	.02^b^
	Knee at night	↓3.0	↓6.3	.06^c^	<.001^b^
**Medication use,** **decrease or increase (%)**					
	Opiate	↓23.2	↓44.3	.02^b^	<.001^b^
	NSAIDs^d^	↓9.2	↓12.8	.08^c^	.06^c^
	Acetaminophen	↑14.6	↑21.0	<.001^b^	<.001^b^
	Gabapentin	↑4.6	↓76.3	.71	<.001^b^
**Acceptable pain,** **decrease or increase (%)**					
	Knee at rest	↓31.3	↓20.3	.11	.25
	Knee during activity	↓17.2	↑31.1	.40	.38
	Knee at night	↓21.1	↑36.4	.21	.25
Experience with the executed recommended exercises—exactly enough, decrease or increase (%)		↓33.1	↓8.7	.02^b^	.67

^a^VAS: visual analog scale.

^b^Significant difference (*P*<.05).

^c^Trend (.05<*P*<.10).

^d^NSAIDs: nonsteroidal anti-inflammatory drugs.

**Table 4:** Findings in the PainCoach group, active PainCoach subgroup, and control group 1 month after surgery.

		1. PainCoach (n=38)	2. Active PainCoach (n=19)	3. Control (n=33)	*P* value (1 vs 3)	*P* value (2 vs 3)
**VAS^a^ pain, median (IQR^b^)**						
	Knee at rest	11.5 (5.0-20.8)	11.5 (4.3-18.8)	10.0 (5.0-25.0)	.77	.53
	Knee during activity	14.0 (7.0-28.8)	12.5 (9.3-26.3)	15.0 (8.0-35.0)	.49	.59
	Knee at night	15.0 (7.0-33.0)	15.0 (5.0-33.0)	15.0 (7.0-27.8)	.79	.89
**Acceptable pain,** **n (%)**						
	Knee at rest	31 (96.9)	16 (100.0)	28 (96.6)	>.99	>.99
	Knee during activity	30 (93.8)	15 (93.8)	25 (86.2)	.41	.64
	Knee at night	26 (81.3)	14 (87.5)	26 (89.7)	.48	>.99
KOOS-PS^c^, mean (SD)		36.5 (10.5)	33.5 (8.4)	39.6 (9.8)	.24	.04^d^
OKS^e^, mean (SD)		28.4 (8.4)	29.9 (9.1)	26.8 (6.2)	.42	.18
EQ-5D^f^ descriptive system, median (IQR)		0.775 (0.693-0.843)	0.811 (0.775-0.857)	0.775 (0.651-0.811)	.34	.11
EQ VAS^g^, median (IQR)		80.0 (70.0-90.0)	83.5 (70.0-90.0)	80.0 (65.5-89.5)	.56	.32

^a^VAS: visual analog scale.

^b^IQR: interquartile range.

^c^KOOS-PS: Knee Injury and Osteoarthritis Outcome Score–Physical Function Short-form.

^d^Significant difference (*P*<.05).

^e^OKS: Oxford Knee Score.

^f^EQ-5D: EuroQol-5 Dimensions.

^g^EQ VAS: EuroQol visual analog scale.

### Other Pain Medication Use, Pain Acceptance, and Experience With Executed Recommended Exercises

In the PainCoach group, there was a statistically significant 14.6% increase in acetaminophen use (95% CI 8.2-21.3; *P*<.001) and no statistically significant differences in NSAID use and gabapentin use when compared with the findings in the control group during the first 2 weeks at home (Table 3). Overall pain medication use was below the advised maximum in both groups. Pain acceptance was 86.5% at rest, 86.5% during activity, and 79.4% at night in the PainCoach group and was 90.4% at rest, 88.6% during activity, and 83.0% at night in the control group, without statistically significant differences between the two groups. Regarding experience with executing recommended exercises, the PainCoach group had statistically significant 33.1% reduced experience with executing exactly enough exercises when compared with the findings in the control group (69.7% vs. 77.5%; 95% CI −52.0 to −6.7; *P*=.02) (Table 3). At 1 month after surgery, no statistically significant differences were found when comparing both groups (Table 4).

Adjusted analyses comparing the active PainCoach subgroup with the control group showed statistically significant 21.0% increased acetaminophen use in the active PainCoach subgroup (95% CI 12.6-30.0; *P*<.001) during the first 2 weeks at home. Additionally, the active PainCoach subgroup had statistically significant 76.3% decreased gabapentin use when compared with the findings in the control group (mean 0.1 [SD 0.3] tablets a day vs. 0.4 [SD 1.0] tablets a day; 95% CI −86.0 to −59.8; *P*<.001) (Table 3). In the active PainCoach subgroup, pain acceptance was 88.4% at rest, 90.9% during activity, and 87.4% at night. Regarding pain acceptance and experience with executing recommended exercises, no statistically significant differences were found between the active PainCoach subgroup and control group (Table 3). One month after surgery, the mean KOOS-PS was significantly lower in the active PainCoach subgroup (33.5 [SD 8.4]) than in the control group (39.6 [SD 9.8]) (*P*=.048) (Table 4).

### PainCoach App Use

Among 28 patients who provided appropriate responses, 25 (89%) reported ease of app use, 22 (79%) found that the app added value, and 22 (79%) would recommend the app to friends and family. The PainCoach app was used 12 (IQR 4.5-22.0) times per patient on 7 (IQR 4.0-9.0) days at home. The number of patients with at least one entry in the PainCoach app ranged from 11 (30%) to 26 (70%) per day at home (Multimedia Appendix 2). The app was most frequently used between 9 and 10 am and mostly for advice on bearable pain.

## Discussion

### Principal Findings

This study aimed to determine the effects of an eHealth app, the PainCoach app, on pain control and opiate use in patients who underwent TKR during the first 2 weeks at home after surgery. The hypothesis was that the app would decrease pain and opiate use. As indicated by the main findings, there was no statistically significant difference in pain scores between the two groups and opiate use was significantly reduced by 23.2% in the PainCoach group when compared with the finding in the control group. In the active PainCoach subgroup, however, pain during activity and at night significantly decreased 4.1 and 6.3 times faster, respectively, and opiate use significantly reduced by 44.3% when compared with the findings in the control group.

Overall, low pain scores and high levels of pain acceptance were found in this study. Only 21% (8/38) of patients in the PainCoach group and 30% (10/33) in the control group classified their pain as severe during one or more days at home. Other studies have stated that the most painful period after TKR surgery was the initial period at home, with 23%-30% of patients rating their average pain as severe [40,41]. Aside from the use of modern LIA techniques and a step-wise pain management protocol postoperatively, a possible explanation for the reported low pain and high acceptance scores in this study could be the guidance program that was provided to all patients who underwent TKR in Kliniek ViaSana. As less anxiety is associated with lower pain scores [14,19], the guidance provided might have resulted in less anxiety and therefore lower pain scores. The reported overall low pain scores also probably explain why no difference in pain scores was found between the PainCoach group and control group. Although overall pain scores were low, active use of the PainCoach app resulted in even lower pain scores during activity and at night when compared with the findings in the control group. These findings are in line with the results of a previous study showing that pain decreased by 0.7 points on a scale from 0 to 10 in patients with OA after online “pain coping skills” training [29]. Others have stated that 80% of interactive information is remembered compared with 20% of auditory information and 40% of read information [30,42,43]. As the PainCoach app is an interactive tool, it is logical that active use will result in better use of the pain management strategies provided and subsequently lower pain scores.

Opiate addiction caused 74 deaths in the Netherlands in 2016, and this number is increasing each year [44]. Using the PainCoach app, opiate use reduced by 23.2%, and active PainCoach app use resulted in a further reduction (44.3%). Because of a lack of standardized opiate prescribing protocols in orthopedic surgery, it is difficult to compare the reported amount of opiate use in this study with that in other studies. In one available study, a daily average morphine dose at discharge of 155 (SD 63) mg was prescribed to patients who underwent TKR, which would be the equivalent of 11 tablets per day of the opiate used in this study (oxycodon, 5 mg per tablet) and is far above the average use of 0.4 opiate tablets per day in this study [45]. The low preoperative opiate use of patients in this study might have contributed to the low opiate use after surgery, as preoperative opiate use is a strong predictor for prolonged opiate use after TKR [42,46,47]. With lower opiate use, acetaminophen use was higher, with a 14.6% increase in the PainCoach group and 21.0% increase in the active PainCoach subgroup. It can be concluded that because of the advice provided by the PainCoach app, opiate use was substituted by acetaminophen use. Opiate use was only advised in the presence of severe enough reported pain in the app. Therefore, it is concluded that the app helps to reduce the risk of the adverse effects of opiate use [48,49].

A shorter hospital stay is associated with a higher burden among patients, who need to take responsibility for aftercare shortly after surgery. Recent studies have shown that patients feel uncertain and left alone after discharge, which could increase anxiety and affect their pain coping and subsequent management [50,51]. Patients might need more individualized guidance, and the PainCoach app was developed to satisfy this need. The app scored high on usability, likelihood of being recommended to others, and added value. The results of this study show that the PainCoach app is a successful pain management tool, and its active use is recommended for the best effects on pain and opiate use.

To our knowledge, this is the first randomized controlled trial to examine the effects of eHealth with regard to controlling pain and reducing opiate use after TKR. The strengths of this study are that the actual amount of app use was measured and because of the unique download codes adopted, it was not possible for the control group to use the PainCoach app. The shortcomings are that the additional analysis was underpowered and the cost-effectiveness of the PainCoach app was not investigated. Furthermore, as there is no short validated questionnaire in Dutch for measuring pain acceptance, an expert group decided to assess pain acceptance using happy and sad smileys as the best alternative. In the population of this study, opiate use was already low. The app might have a much stronger effect in patient populations where preoperative opiate use is much higher. It is questionable if the PainCoach app is effective in the overall TKR population, as this study investigated the effects in patients having ASA I-II and BMI ≤35, which represent around 80% of the total TKR population [52,53]. Future research should focus on a larger sample size of the total TKR population, determination of the cost-effectiveness of the app, and use of the app in populations that have much higher preoperative opiate use.

### Conclusions

The use of the PainCoach app contributes to reduced opiate use in the initial period at home after TKR. Active use of this app leads to further reduction in opiate use and improved pain control.

## Appendix

Multimedia Appendix 1 Pain management protocol.

Multimedia Appendix 2 Content and use of the PainCoach app.

Multimedia Appendix 3 CONSORT‐EHEALTH checklist (V 1.6.1).

## Abbreviations

ASA: American Society of Anesthesiologists
BMI: body mass index
eHealth: electronic health
EQ: EuroQol
EQ-5D: EuroQol-5 Dimensions
EQ-5D-3L: EuroQol-5 Dimensions 3-level version
EQ VAS: EuroQol visual analog scale
IQR: interquartile range
KOOS-PS: Knee Injury and Osteoarthritis Outcome Score–Physical Function Short-form
LIA: local infiltration anesthesia
NSAIDs: nonsteroidal anti-inflammatory drugs
TKR: total knee replacement
VAS: visual analog scale
