# Supplementary material for: Effectiveness of a Mobile eHealth App in Guiding Patients in Pain Control and Opiate Use After Total Knee Replacement: Randomized Controlled Trial
Source: JMIR Mhealth Uhealth. 2020 Mar 13;8(3):e16415. doi: 10.2196/16415 (PMC7101497; doi:10.2196/16415)
Supplement: Multimedia Appendix 1 [file mhealth_v8i3e16415_app1.pdf]

Appendix 1 – Pain Management Protocol

Table 1. Pain Management Protocol

	Medication	Intake	Dosage
**Pre-operative**	Gabapentin	2 hours before surgery 2 tablets	600 mg
	Acetaminophen (Paracetamol)	2 hours before surgery 2 tablets	1000 mg
	NSAID[a] (Diclofenac)	2 hours before surgery 1 tablet	50 mg
**Per-operative**	LIA[b]	*Ropivacaine 2 mg/ml and Adrenalin 1 mg/ml at a total volume of 100 ml*<br>LIA 1: injection of 2 x 20 ml Ropivacaine 2 mg/ml with Adrenalin in the posterior joint capsule and both collateral ligaments before the prosthesis was placed.<br>LIA 2: After placement of the prosthesis, 2 x 20 ml Ropivacaine 2 mg/ml with Adrenalin injections along the edges of the tibia, in the capsule and in fat and soft tissue around the joint were injected.<br>LIA 3: Inject 20 ml Ropivacaine 2 mg/ml without Adrenalin in subcutaneously layers before the wound was stitched.	
**Post-operative during admission**	Acetaminophen (Paracetamol)	2 tablets, 4 times per day	500 mg
	NSAID[a] (Diclofenac)	1 tablet, 3 times per day	50 mg
	Gabapentin	Day of surgery 10.00 pm<br>Day after surgery 08.00 am<br>Thereafter at indication	300 mg<br>300 mg
		If necessary (NRS > 4)	
	Opiate (Oxynorm / oxycodon)	Maximum of 1 tablet, 3 times per day	5 mg
**Post-operative at home - *Usually 1 or 2 nights after surgery***	Acetaminophen (Paracetamol)	2 tablets, 4 times per day (until day 14 after surgery)	500 mg
		In presence of pain	
	NSAID[a] (Diclofenac)	1 tablet, 3 times per day (until day 7 after surgery)	50 mg
	Opiate (Oxynorm / oxycodon)	Maximum of 1 tablet, 3 times per day	5 mg
	Gabapentin	At indication on doctor's prescription	

[a]NSAID: non-steroidal anti-inflammatory drug.

[b]LIA: local infiltration anesthesia.
