# Supplementary material for: Effectiveness of a Mobile eHealth App in Guiding Patients in Pain Control and Opiate Use After Total Knee Replacement: Randomized Controlled Trial
Source: JMIR Mhealth Uhealth. 2020 Mar 13;8(3):e16415. doi: 10.2196/16415 (PMC7101497; doi:10.2196/16415)

Appendix 2 – Content and use of the PainCoach app

Table 1. Short schematic overview PainCoach app content.

Post-operative days	Inputted pain level	Pain medication advice	Other advice
1-2	No pain	Acetaminophen (Paracetamol): 2 tablets, 4 times per day. Dosage 500 mg. NSAID (Diclofenac)[a]: 1 tablet, 3 times per day. Dosage: 50 mg.	Execute exercises. Rest between exercises: immobilising the operated leg.
	Bearable pain	Acetaminophen (Paracetamol): 2 tablets, 4 times per day. Dosage 500 mg. NSAID (Diclofenac)[a]: 1 tablet, 3 times per day. Dosage: 50 mg.	Execute exercises. Use ice and heat packs. Rest between exercises: immobilising the operated leg.
	Unbearable pain	Acetaminophen (Paracetamol): 2 tablets, 4 times per day. Dosage 500 mg. NSAID (Diclofenac)[a]: 1 tablet, 3 times per day. Dosage: 50 mg. Opiate (Oxynorm / oxycodon): Maximum of 1 tablet, 3 times per day. Dosage: 5 mg.	Execute exercises without forcing. Contact physiotherapist for advice. Use ice and heat packs. Rest between exercises: immobilising the operated leg.
	Untenable pain	Call the clinic.	Execute exercises without forcing. Contact physiotherapist for advice. Use ice and heat packs. Rest between exercises: immobilising the operated leg.
3-7	No pain	Acetaminophen (Paracetamol): 2 tablets, 4 times per day. Dosage 500 mg.	Execute exercises. Rest between exercises: immobilising the operated leg.
	Bearable pain	Acetaminophen (Paracetamol): 2 tablets, 4 times per day. Dosage 500 mg. NSAID (Diclofenac)[a]: 1 tablet, 3 times per day. Dosage: 50 mg.	Execute exercises. Use ice and heat packs. Rest between exercises: immobilising the operated leg.
	Unbearable pain	Acetaminophen (Paracetamol): 2 tablets, 4 times per day. Dosage 500 mg. NSAID (Diclofenac)[a]: 1 tablet, 3 times per day. Dosage: 50 mg. Opiate (Oxynorm / oxycodon): Maximum of 1 tablet, 3 times per day. Dosage: 5 mg. Call the clinic for gabapentin at indication on doctor's prescription.	Execute exercises without forcing. Contact physiotherapist for advice. Use ice and heat packs. Rest between exercises: immobilising the operated leg.

	Untenable pain	Call the clinic.	Execute exercises without forcing.<br>Contact physiotherapist for advice.<br>Use ice and heat packs.<br>Rest between exercises:<br>immobilising the operated leg.
8-14	No pain	Acetaminophen (Paracetamol):<br>2 tablets, 4 times per day.<br>Dosage 500 mg.	Execute exercises.<br>Rest between exercises:<br>immobilising the operated leg.
	Bearable pain	Acetaminophen (Paracetamol):<br>2 tablets, 4 times per day.<br>Dosage 500 mg.	Execute exercises.<br>Use ice and heat packs.<br>Rest between exercises:<br>immobilising the operated leg.
	Unbearable pain	Acetaminophen (Paracetamol):<br>2 tablets, 4 times per day.<br>Dosage 500 mg.<br>NSAID (Diclofenac)[a]: 1 tablet,<br>3 times per day. Dosage: 50 mg.<br>Opiate (Oxynorm / oxycodon):<br>Maximum of 1 tablet, 3 times per day. Dosage: 5 mg.<br>Call the clinic for gabapentin at indication on doctor's prescription.	Execute exercises without forcing.<br>Contact physiotherapist for advice.<br>Use ice and heat packs.<br>Rest between exercises:<br>immobilising the operated leg.
	Untenable pain	Call the clinic.	Execute exercises without forcing.<br>Contact physiotherapist for advice.<br>Use ice and heat packs.<br>Rest between exercises:<br>immobilising the operated leg.

[a]NSAID: non-steroidal anti-inflammatory drug.

Figure 1. Screenshots of PainCoach app showing how the PainCoach app works and how the content was presented to the patients.

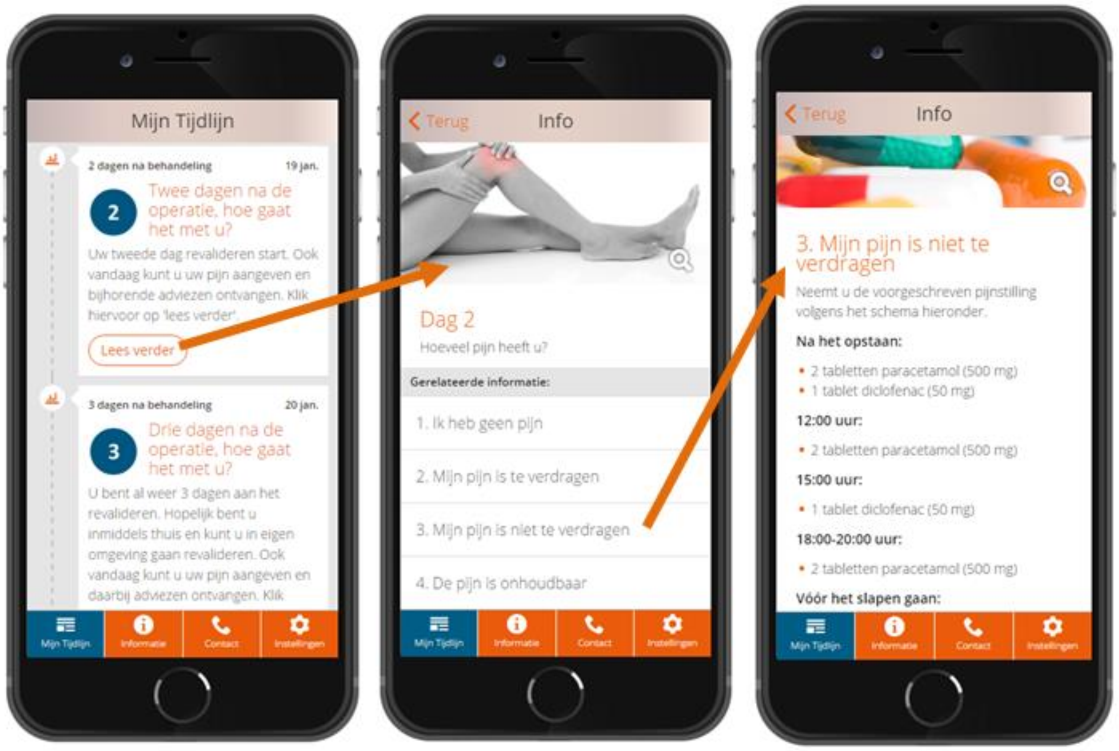

Figure 2. Patients with at least one entry in the PainCoach app per day at home.

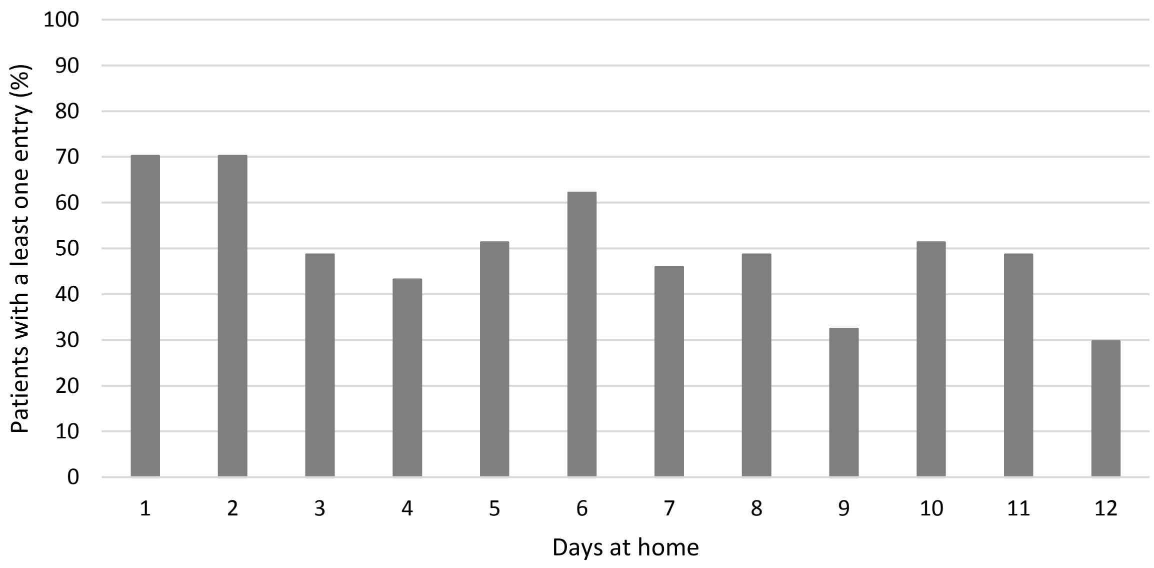

Figure 3. Total days at home patients entered the PainCoach app.

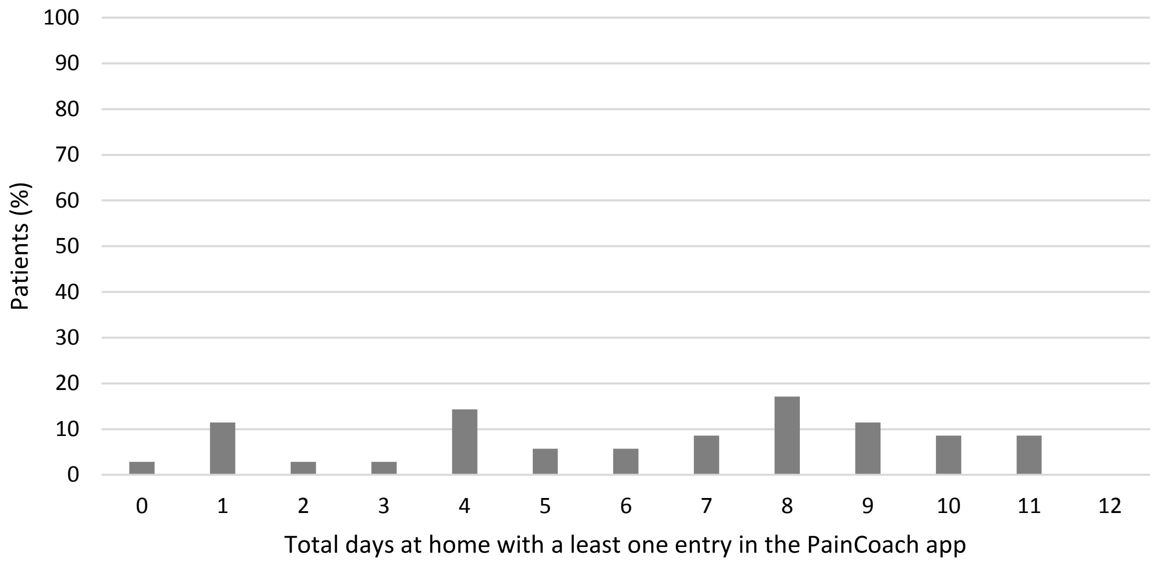

Supplement: Multimedia Appendix 2 [file mhealth_v8i3e16415_app2.pdf]
